# The Role of Metformin in Ovarian Cancer: Does Metformin Increase Survival in Ovarian Neoplasm?

**DOI:** 10.7759/cureus.13100

**Published:** 2021-02-03

**Authors:** Maimuna F Ahmed, Ghid Kanaan, Jihan A Mostafa

**Affiliations:** 1 General Medicine, California Institute of Behavioral Neurosciences & Psychology, Fairfield, USA; 2 Pediatrics, California Institute of Behavioral Neurosciences & Psychology, Fairfield, USA; 3 Psychiatry, California Institute of Behavioral Neurosciences & Psychology, Fairfield, USA

**Keywords:** ovarian neoplasm, metformin, ovary cancer, biguanides, dimethylbiguanides, female genital neoplasm, epithelial ovarian cancer

## Abstract

The role of metformin in ovarian cancer (OC) remains a topic of research and open discussion. Because OC has a high mortality rate for various reasons, finding a solution is vital. Although metformin has demonstrated a high level of evidence in preventing and increasing survival in other cancers, its role in OC is still not proven. This review highlights the function of metformin as an antineoplastic agent in OC and its effect on overall survival, progress-free survival, and recurrence-free survival. We conducted a literature search in the PubMed database using the medical subject heading keywords, ovarian neoplasm and metformin. The search yielded 94 articles, of which 86 remained after including only English language articles. Finally, 50 articles published between 1997 and 2020 were reviewed. We recommend more randomized controlled trials in the future to determine the safety and efficacy of metformin in OC.

## Introduction and background

Cancer is the second leading cause of death worldwide, with ovarian cancer (OC) being the fifth leading cause of cancer-related death, affecting about seven per 100,000 women annually. According to data from 2015-2017, about 1% of women will be diagnosed with OC at some point during their lifetime [1]. In 2018, the financial burden of cancer care in the United States was about $150.8 billion. These numbers are expected to increase, with the rising cost of cancer medications being one of the factors [1].

OC is a rare gynecological cancer. It arises from the epithelial, sex cord stromal, or germ cells, as well as the endometrium. Epithelial ovarian cancer (EOC) is the most common type with a high mortality as it is diagnosed late for various reasons. First, there is no recommended screening test for women without symptoms and those at low risk of developing OC. Although the availability of transvaginal ultrasound and cancer antigen 125 (CA-125) for screening women with moderate-to-high risk of OC increased testing and prophylactic surgeries, it failed to lower the number of deaths caused by OC [2]. Second, OC presents with non-specific symptoms. Furthermore, patients can remain asymptomatic or an abdominal mass can be discovered during a routine examination. The treatment of choice for OC is surgery. In advanced cases, tumor debulking followed by adjunctive therapy is suggested for improved efficacy [3]. However, in advanced ovarian neoplasms, most patients have a recurrence of ovarian neoplasm [4]. Unfortunately, the currently available systemic therapy for recurrent OC, such as cytoreductive surgery followed by combination chemotherapy with taxane and platinum, has limited efficacy and increased toxicity [5]. Studies have suggested paclitaxel for prolonged progression-free survival (PFS), but its clinical significance has not been proven [6]. While some chemotherapy treatments offer improved PFS, they can be cost-prohibitive for patients [7].

Metformin, a biguanide, was first discovered in the 1920s as an anti-diabetic medication. Since then, metformin has improved mortality rates in diabetes, cancer, obesity, polycystic ovary syndrome, and metabolic syndrome [8]. Evan et al. suggested that metformin may reduce cancer incidence in type two diabetes mellitus (T2D) [9]. Metformin has shown significant survival outcomes in cancers such as colorectal cancer, prostate cancer [10], and hormone receptor-positive breast tumors [11]. Various studies have show an association between metformin and improved prognosis; however, investigations have not been able to fully establish the effect of metformin in OC.

This traditional review focuses on the mechanism of action of metformin and its role in overall survival (OS), PFS, and recurrence-free survival (RFS) in OC. We conducted a literature search in the PubMed database using the medical subject heading keywords, ovarian neoplasm and metformin. This search yielded 94 articles, of which 86 remained after including only English language articles. Finally, 50 articles published between 1997 and 2020 were reviewed. Enhanced knowledge about the mechanism of action and its effect on survival can help find better prognosis solutions in OC patients. Metformin can be a safe, cost-effective, and less toxic option in reducing OC-related mortality. Here, we review preclinical and clinical studies to determine if metformin can be a potential lifesaver in OC.

## Review

Mechanism of action of metformin

The antidiabetic effect of metformin is through an enzyme called adenosine monophosphate-activated protein kinase (AMPK). Metformin reduces hepatic gluconeogenesis and promotes glucose uptake by muscles via AMPK activation, thus lowering blood glucose and insulin levels and reducing insulin resistance, with the latter being a risk factor for OC [12,13]. The antineoplastic effect of metformin in OC is through AMPK and various other mechanisms, which we will explore here.

Metformin and Mitochondria

Metformin acts as an anticancer drug through AMPK-dependent and independent pathways. Metformin primarily targets mitochondria and affects mitochondria-related energy status, nucleotide metabolism, and oxidation state [14]. It inhibits mitochondrial electron transport chain (ETC) complex one, produces energy stress, depletes adenosine triphosphate (ATP), raises adenosine monophosphate (AMP)/ATP ratio, and activates AMPK, which, in turn, inhibits anabolic processes for cell growth [15]. Moreover, energy stress induced by ETC inhibition increases sirtuin three (SIRT3) expression. Increased SIRT3 expression promotes mitochondrial dysfunction and aggravates energy stress and cell oxidative damage, leading to apoptosis, a cell death process. Additionally, elevated reactive oxygen species (ROS) induced by energy stress activates the tuberous sclerosis protein complex two (TSC2) pathway, which suppresses mammalian target of rapamycin (mTOR), thus inhibiting cell growth and activating autophagy [16].

Metformin and Metabolic Reprogramming

Coordination alteration between cellular stimulatory signals generated by nutrients/growth factors and inhibitory signals generated by intracellular/extracellular stresses causes cancer. mTOR regulates stimulatory signals and p53 regulates inhibitory signals. Metformin inhibits mTOR via liver kinase B one (LKB1)-mediated AMPK activation [17]. Metformin also activates AMPK; subsequently, TSC2, followed by p53 activation, inhibits mTOR, consequently inhibiting translation and biogenesis in ribosomes. Inhibiting cell proliferation induces autophagy [18].

Independent of AMPK, metformin decreases the circulating levels of insulin and insulin growth factor one (IGF-1), inhibiting the signaling pathways involved in cell proliferation and growth, as well as impairing OC initiation and progression in vivo [19,20]. The data above can explain the reduction of cancer incidence in a diabetic patient taking metformin.

OC displays significant overexpression of cysteine-rich 61 (Cyr61), which is associated with poor prognosis [21]. Metformin directly inhibits Cyr61 and modulates phosphatidylinositol three-kinase (PI3K)/protein kinase B (Akt)/ mTOR cascade, which is responsible for chemo-resistance and poor prognosis of OC; consequently, cell viability loss, invasion suppression, and apoptosis ensue [22].

Furthermore, cellular myelocytomatosis oncogene (c-MYC) regulates several gene expressions and proteins in the cell cycle, proliferation, division, and growth; hence, c-MYC overexpression increases cell proliferation and growth. Nerve growth factor (NGF) eis responsible for EOC progression by increasing the transcriptional activity of c-MYC, survivin, and vascular endothelial growth factor (VEGF) [23]. Metformin inhibits NGF and lowers the expression of c-MYC, survivin, and VEGF in EOC cells, thus inducing degradation of cyclidin d one, a protein involved in the cell cycle and required for cell progression from cell growth phase one (G1) to DNA synthesis (S) phase, and increasing protein 21 (p21) gene expression, resulting in cell cycle arrest [23-25].

Metformin and Lipids

OC displays high lipid metabolism rate, with increased fatty acid synthase (FAS) expression associated with poor prognosis and survival [26]. Metformin inhibits receptor tyrosine kinase (RTK) in an AMPK-dependent manner, leading to growth inhibition and potentiating the paclitaxel effect in OC. AMPK precludes cholesterol and FAS by inhibiting hydroxy-methylglutaryl-coenzyme-A (HMG-CoA) reductase and acetyl co-A carboxylase (ACC), depriving cells of pivotal molecules for growth and proliferation [27].

Additionally, metformin suppresses migration and chemotaxis in OC by targeting sphingolipid rheostat and promotes apoptosis. Sphingolipid rheostat comprises sphingosine-one-phosphate (S1P) in balance with ceramides and sphingosine kinase one (SPHK1). SPHK1 catalyzes S1P that supports cell growth and proliferation, and hence, tumor progression and survival. Metformin reduces SIP levels via downregulating SPHK1 [28].

Metformin and Glucose Status

Interestingly, cellular glucose status influences metformin action and cytotoxicity. In vivo, metformin shows a significant growth inhibition in normoglycemic cells compared to hyperglycemic ones. This effect is through metformin-induced AMPK activation [27]. Furthermore, hyperglycemic OC cells display increased c-MYC expression, which mediates an increase in glycolysis as a compensation for metabolic changes. This expression is suppressed by metformin in normoglycemic cells, highlighting the role of c-MYC overexpression in metformin resistance even in normoglycemia and the potential utilization of c-MYC to predict the response to metformin [29]. Additionally, metformin creates energy stress, yielding ROS accumulation, which induces mitochondrial damage and endoplasmic reticulum stress via apoptosis signal-regulating kinase 1 (ASK1) and c-Jun N-terminal kinase (JNK) pathway activation, leading to apoptosis in low glucose condition [30].

Metformin and Epigenetic Alteration

Metformin can reduce cancer progression through epigenetic alteration. The trimethylation of lysine 27 on histone H three protein subunit (H3K27me3) is responsible for chemotherapy/cisplatin resistance and cancer progression. Metformin inhibits H3K27me3 via AMPK activation, yielding cytotoxic effects, and reduces cisplatin resistance in vitro and in vivo [31]. Additionally, metformin through AMPK activation inhibits fatty acid synthesis, leading to acetyl-coenzyme-A accumulation, which induces histone acetylation, hence altering gene expression. Metformin induces histone and non-histone protein acetylation in OC cells via AMPK activation [32]. Furthermore, metformin exhibits an anticancer effect via modulation of dicer, an endonuclease in the microRNA (miRNA) axis, which plays a crucial role in cell proliferation, differentiation, metabolic reprogramming, angiogenesis, and apoptosis. Low expression of dicer is associated with a poor prognosis in OC [33].

Metformin and Immunity

OC displays an elevated cluster of differentiation (CD)39/CD73 expression, mediated by hypoxia-inducible factor one-alpha (HIF-1α) expression on myeloid-derived suppressor cells (MDSCs). This expression inhibits T cell activity and protects cancer cells from chemotherapy cytotoxicity. Metformin reduces CD39/CD73 and HIF-1α expression on MDSCs through AMPKα activation and decreases MDSC-mediated immunosuppression [34].

Metformin and Stem Cell Studies

Cancer stem cells (CSCs) are identified by their biomarkers aldehyde dehydrogenase (ALDH+) and CD133 [35]. CSCs initiate primary cancer, display chemotherapy resistance, and correlate with poor survival, especially when both markers are present. Metformin targets OC CSCs and displays an antiproliferative effect by reducing ALDH+ CSCs percentage and inhibiting CSCs sphere formation in an AMPK-dependent and independent manner. Metformin alters DNA methylation of cancer mesenchymal stem cells. Consequently, metformin decreases CA-MSC-mediated stem-ness and chemo-resistance and increases OS. Notably, platinum sensitivity increases in the presence of metformin [36].

**Figure 1:**
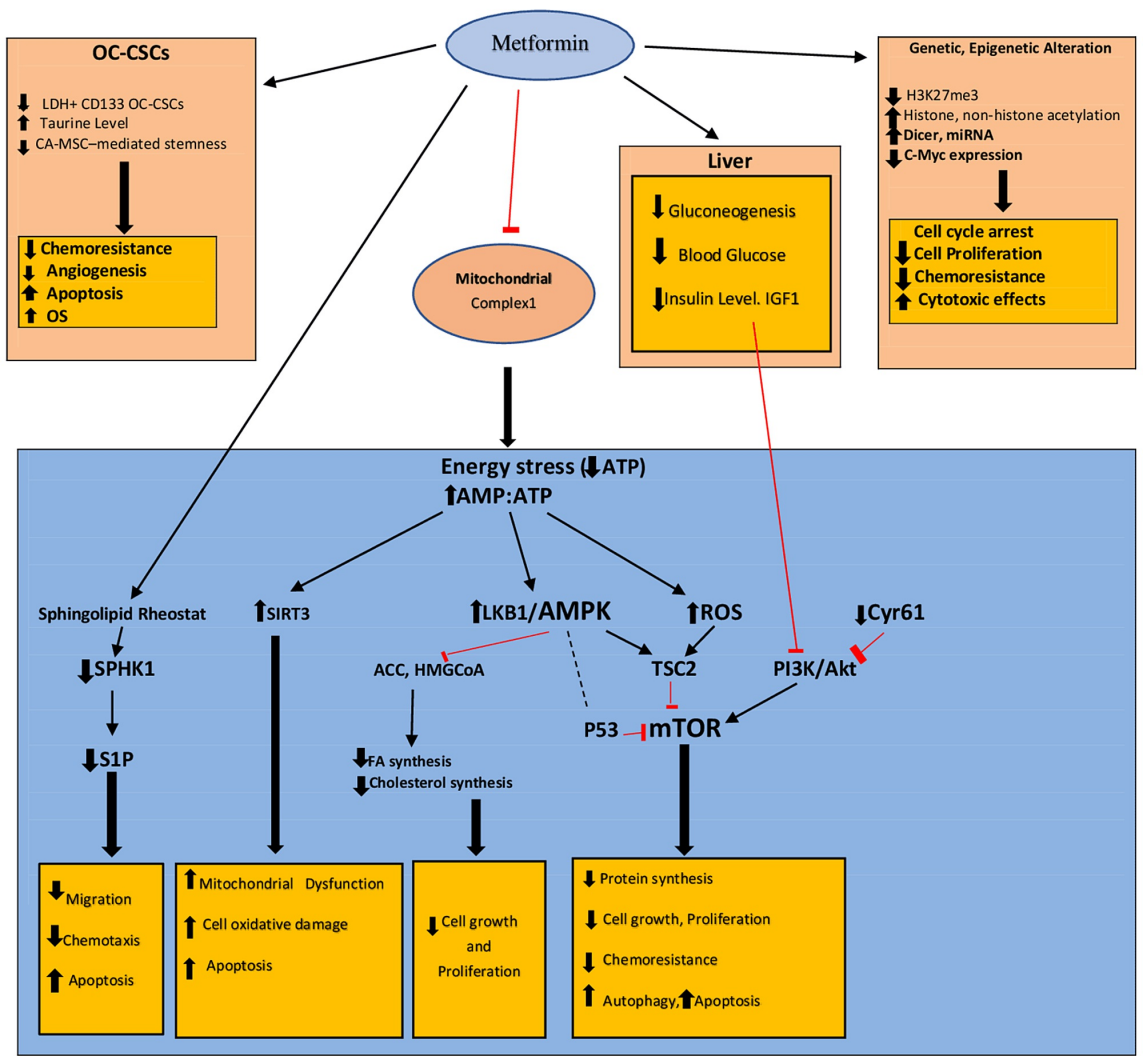
Mechanism of action of metformin in OC by organ, molecule, and pathways. ACC = acetyl co-A carboxylase; ALDH = aldehyde dehydrogenase; AMP = adenosine monophosphate; AMPK = adenosine monophosphate-activated protein kinase; ATP = adenosine triphosphate; cMYC = cellular myelocytomatosis oncogene; CA-MSC = carcinoma-associated mesenchymal stem cells; CSCs = cancer stem cells; Cyr 61 = cysteine-rich 61; CD39/CD73 = cluster of differentiation; FA = fatty acid; H3K27me3 = trimethylation of lysine 27 on histone H three protein subunit; HMG-CoA = hydroxy-methylglutaryl-coenzyme-A; IGF1 = insulin-like growth factor one; LKB1 = liver kinase B one; miRNA = microRNA; mTOR = mammalian target of rapamycin; OC = ovarian cancer; OS = overall survival; P53 = protein53; PI3K/Akt = phosphatidylinositol three-kinase/protein kinase B; ROS = reactive oxygen species; SIRT3 = sirtuin 3; SPHK1 = sphingosine kinase one; S1P = sphingosine-one-phosphate; TSC2 = tuberous sclerosis protein complex two Red lines = inhibition; black thin arrows = activation

Metformin lowers chemo-resistance and improves chemo-sensitivity in CSCs by elevating taurine levels. The introduction of taurine with cisplatin-paclitaxel increases cell death compared with cisplatin-paclitaxel used alone. Long-term use of metformin in low doses increases taurine levels, which reduces chemo-resistance [37]. Metformin, as an adjuvant to bevacizumab, inhibits angiogenesis in the CSCs [38]. Figure 1 summarizes the mechanism of action.

Metformin clinical studies

Overall Survival

Observational studies have shown the association of metformin with increased OS in OC. Currie et al. observed all-cause and cancer-specific mortality in diabetic and non-diabetic patients. The results showed lower mortality in T2D patients on the metformin regimen than non-diabetic patients [39]. Another study showed that OS was statistically significant at a median of 138 months among T2D patients on metformin compared to 42 months in non-diabetics and 35 months in the non-metformin group; most patients were on standard chemotherapy [40]. A study compared OC patients on metformin (cases) to non-diabetics in OC cohort and EOC cohorts. The two groups had the same age participants, same year of diagnosis, underwent surgical cytoreduction, and platinum-based chemotherapy. The median survival was five and a half years in cases compared to four years in controls in the OC cohort. The five-year survival rate was also higher in OC cohort cases. After adjustment, the multivariant analysis showed only the grade of OC and metformin usage remained independent predictors of survival. EOC cases had significantly better five-year disease-specific survival, 67% than controls (47%) [41]. Wang et al. retrospectively observed 568 women diagnosed with OC for three years. A total of 104 out of 568 patients were diabetics, of which were using metformin from the baseline and 22 stopped using metformin. The results showed that diabetic patients using metformin had significantly longer OS than other groups. After adjustments, metformin continued to show a significantly lower risk of disease relapse and disease-related death in the group using metformin from the baseline [42]. However, another study showed no statistical differences in mortality from OC or from other causes between the metformin and other antidiabetic medications [43]. The results from systemic reviews and meta-analysis are inconsistent. While some reported a positive effect of post-diagnosis metformin use on OC incidence, prognosis, and survival outcomes in diabetic patients, others indicated that metformin might not be associated with survival benefits [44-47].

Progress-Free Survival

PFS is calculated from the date of diagnosis until the date of OC recurrence or death. A study reported statistically significant five-year PFS at 51% for diabetic patients on metformin, 23% in non-diabetic patients, and 8% in diabetic patients not using metformin, which was maintained after adjustment [40]. Wang et al. observed a significantly longer PFS in the metformin group at 40 months compared to the non-metformin group at 18.2 months, the discontinued group at 28 months, and the non-diabetic group 23.3 months [42]. A study reported that stage IIC/III disease had a median PFS of only 18.3 months, while patients with stage IV disease had 14.8 months, followed up for 45 months [36]. Another study determined PFS was not significant in patients taking metformin compared to non-metformin patients [48]. However, a systemic review reported metformin has an effect on PFS in OC patients [49].

Recurrence-Free Survival

RFS is the period free from evidence of reappearance of cancer by clinical examination, computed tomography, or ultrasound scan, or an increase in CA-125 two times or more the upper limit of 70 U/mL. A study defined six months as RFS and showed metformin group with the highest percentage of RFS; however, it was not statistically significant [40]. Another study reported a significant median RFS of 32 months for cases and 22 months for controls [41]. Bar et al. observed a cohort with a median age of 62.5 diagnosed with OC from stage I to IV. Approximately 20% of the patients received neoadjuvant chemotherapy, 15.4% had T2D at the time of OC diagnosis, and about 8.5% used metformin for at least one year. Overall, 70.6% of the patients experienced disease recurrence during the follow-up period. The median RFS was 25.7 months. Multivariate analysis, including age at diagnosis, stage at diagnosis, use of neoadjuvant chemotherapy, the presence of T2D, hypertension, and the use of the different drug classes, was performed. Stage and the presence of T2D were associated with a shorter RFS, while metformin use was associated with a significantly prolonged RFS [50]. Wang et al. reported significantly increased RFS when diabetic patients on metformin were compared to patients with T2D but not on metformin [42]. Brown et al. determined 59.3% of the patients had RFS at 18 months [36].

Metformin as an Adjuvant to Chemotherapy in Non-diabetics

Zheng et al. evaluated the efficacy of metformin as an adjuvant to chemotherapy. The disease-free survival of patients on metformin versus those not on metformin was 29 versus 26 months. The improvement trend was evident, though the p-values were insignificant. There were no dropouts due to adverse events. Although metformin effectively reduced IGF-1 levels, the study could not demonstrate its effectiveness due to lack of insulin sensitivity measurement, small sample size, and short follow-up [48].

In a phase two trial, EOC was treated with metformin before debulking surgery, followed by adjuvant chemotherapy plus metformin or chemotherapy and metformin, as well as interval debulking surgery, adjuvant chemotherapy plus metformin. Median OS was 57.9 months, by stage IIC/III disease was 58.0 months, and 22 months for patients with stage IV disease [36]. The results are encouraging but limited because this was a non-randomized trial with a small sample size. Table 1 lists few ongoing trials investigating the effects of metformin in OC.

**Table 1:** Ongoing trials related to metformin in ovarian cancer.

Condition; NCT	Intervention	Title	Primary purpose
Advanced stage ovarian cancer; NCT02437812	Metformin, paclitaxel, carboplatin	A phase II, open-label, non-randomized, pilot study of paclitaxel, carboplatin and oral metformin for patients newly diagnosed with stage II-IV epithelial ovarian, Fallopian tube or primary peritoneal carcinoma	Safety, toxicity, and progression-free survival
Advanced (stage IIIa-IV) ovarian cancer of the histologic subtype high-grade serous carcinoma (HGSOC); NCT03378297	Metformin vs acetylsalicylic acid vs olaparib vs letrozole	A phase 0 randomized window-of-opportunity study of novel and repurposed therapeutic agents in women triaged to primary surgery for advanced epithelial ovarian cancer in stages IIIa-IV	Changes in the expression of biomarkers
Advanced stage III/IV ovarian, Fallopian tube, and primary peritoneal cancer; NCT02122185	Metformin in conjunction with chemotherapy followed by metformin maintenance therapy	A randomized placebo controlled phase II trial of metformin in conjunction with chemotherapy followed by metformin maintenance therapy in advanced stage ovarian, Fallopian Tube and primary peritoneal cancer	Progression-free survival

## Conclusions

This review aimed to determine the mechanism of metformin’s action in OC and its effect on survival in diabetic and non-diabetic patients. Preclinical and clinical studies have shown that metformin targets several cell molecules and pathways at various levels and exhibits antiproliferative effect, inhibits growth and migration, increases chemo-sensitivity, and induces apoptosis and autophagy. Although OS was statistically significant, some studies showed no association of metformin with OS. A systematic review article emphasized significant increase in PFS. Positive results in RFS have shown statistical significance consistently. The results of studies with metformin use as an adjuvant have been encouraging but limited due to not being RCTs. Metformin could be a non-toxic, safe, and effective way for improving survival in high mortality OC as it has already demonstrated significant benefit in other extensively studied cancers and to some extent in prospective trials related to OC.

The current level of evidence of metformin in OC is moderate and inconsistent as there is a lack of RCTs. The meta-analysis conducted so far included only observational studies. The level of evidence could be improved by conducting more RCTs targeting metformin’s efficacy in non-diabetic OC patients with longer follow-ups. An inexpensive and less toxic medication could change the treatment approach for OC in the future.
